# Knowledge and practices of dental trauma by Greek school nurses: a questionnaire-based cross-sectional study

**DOI:** 10.1007/s40368-026-01191-1

**Published:** 2026-03-05

**Authors:** Ioannis Tranoulis, Konstantinos Kodonas, Anastasia Fardi, Myron Bitsakis, Christos Gogos

**Affiliations:** 1 Department of Endodontology, School of Dentistry, Aristotle University of Thessaloniki, Thessaloniki, Greece; 2 Department of Dentoalveolar Surgery, Implantology and Oral Radiology, School of Dentistry, Aristotle University of Thessaloniki, Thessaloniki, Greece

**Keywords:** Traumatic dental injuries, Knowledge, Management, School nurses

## Abstract

**Purpose:**

This study aims to evaluate the knowledge and first-aid practices of Greek school nurses regarding dental trauma.

**Methods:**

A cross-sectional study, utilizing a researcher-developed questionnaire based on the relevant literature, was conducted. Questions about demographic characteristics, work experience, and knowledge of first aid for dental injuries were included*.* Three dental trauma case scenarios of anterior teeth, including crown fracture, lateral luxation, and avulsion were included. The results were statistically analyzed using SPSS at *p* < 0.05%.

**Results:**

A total of 136 school nurses from various regions of Greece responded. The majority of participants (77.2%) had worked as school nurses for fewer than 5 years, and fewer than 35% of the respondents received any training related to dental trauma. When confronted with a crown fracture, most indicated that the tooth was permanent, and 60.3% would place the tooth fragment in liquid for preservation. In the case of lateral luxation, the most common response was to contact the child’s parents. For avulsion, half of the respondents were unaware of the importance of timing; the majority would choose an appropriate transfer medium and refer the patient to a dentist.

**Conclusions:**

Greek school nurses have limited capability to manage dental trauma cases, and their current level of knowledge is insufficient. Current management of dental trauma in schools often deviates from recommended guidelines.

**Supplementary Information:**

The online version contains supplementary material available at 10.1007/s40368-026-01191-1.

## Introduction

Traumatic dental injuries (TDIs) are one of the most important oral health problems in childhood; 25% of all school children may experience dental trauma in permanent teeth (Levin et al. 2020; Saikiran et al. 2022). In Greece, despite the limited data available, the incidence of dental trauma in children aged 5–12 years has been estimated to be 11%—the most common types are crown fracture, followed by lateral luxation, extrusive luxation, crown and root fractures, concussion and subluxation, intrusion, and avulsion (Lygidakis et al. 1998).

The school environment is considered the second most common setting, after the home, where traumatic TDIs may occur, as children spend a substantial portion of their day there (Tzimpoulas et al. 2020; Al-Khalifa et al. 2022; Alharbi et al. 2023). Falls, playtime, and sports activities are the leading causes of TDIs at school (Petti et al. 1996; Patnana et al. 2021; Glendor 2008; Azami-Aghdash et al. 2015). Consequently, parents and schoolteachers are often the first responders responsible for initial TDI management (Al-Khalifa et al. 2022). However, limited awareness of dental trauma management is evident amongst parents, teachers, and other adults likely to encounter such injuries (Al-Khalifa et al. 2022; Aminu et al. 2023; Alharbi et al. 2023; Świątkowska et al. 2018; Kaul et al. 2016; Vashishtha et al. 2016). Recent systematic reviews also indicate that awareness and knowledge of TDIs amongst parents, teachers, and coaches remain limited (Tewari et al. 2020; Tewari et al. 2021a, b). More specifically, many individuals are unable to identify the type of tooth involved (Kaul et al. 2016) or are unaware of the correct handling and storage procedures for avulsed or fractured teeth (Vashishtha et al. 2016). Similar gaps in knowledge have been observed amongst Greek primary school teachers (Tzimpoulas et al. 2020). Such a lack of preparedness may delay appropriate first-aid responses, which are crucial for improving the prognosis of traumatized teeth through timely intervention (Andreasen et al. 2002).

In 1893, Lilian Ward, a progressive nurse, introduced nursing care into schools. Today, school nurses provide immediate care for students who become ill or injured during school hours, playing a crucial role in promoting and maintaining the health and well-being of the entire school community (Guarinoni and Dignani 2021). Since dentists may not be available in school communities for immediate care, school nurses may play a vital role in providing first aid, reassuring the child, informing parents, and facilitating quick referral to a paediatric dentist when necessary (Baginska et al. 2012, 2016; Al Sari et al. 2019).

Many studies have examined healthcare professionals’ knowledge of dental trauma in relation to the International Association of Dental Trauma (IADT) guidelines (Yeng et al. 2020; Tewari et al. 2021a, b; Coşkun et al. 2021; Carrion-Ruiz et al. 2024; Bourguignon et al. 2020; Andersson et al. 2020). Although these guidelines are primarily directed toward dental professionals, such as dentists and dental specialists, the IADT–ASD (Academy for Sports Dentistry) prevention guidelines are generally more applicable to physicians and nurses (Tewari et al., 2024). However, most research has concentrated on avulsion of permanent teeth, because healthcare providers are generally familiar with it, and its prompt treatment significantly influences the outcome of the injured tooth (Tewari et al. 2021a, b). As a result, there is limited literature on managing crown fractures and luxations. In advance, the proficiency of school nurses to provide first aid on TDI has not yet been systematically evaluated, despite their role as first responders in school-based emergencies. Moreover, studies regarding school nurses’ knowledge of TDIs are rare (Baginska et al. 2012, 2016; Al Sari et al. 2019).

In Greece, school nurses are legally responsible for managing emergency health incidents, such as TDIs, within the school communities. However, school nursing remains an underdeveloped and inconsistently regulated field, with school nurses still lacking both specialized recognition and standardized training requirements (Baginska et al. 2012, 2016; Al Sari et al. 2019). The present cross-sectional study aims to assess the knowledge and first-aid practices for TDI by school nurses in Greece.

## Materials and methods

### Study design

An anonymised electronic multiple-choice questionnaire, using the IADT guidelines (Bourguignon et al. 2020; Andersson et al. 2020), was created, using the EUSurvey site (https://ec.europa.eu/eusurvey) to ensure the anonymity of the respondents and the protection of their personal data. This study was approved by the Human Research Ethics Committee of Dental School, Aristotle University of Thessaloniki, Greece (Reference Number: 228/21-03-2024).

### Form of the questionnaire

After reviewing relevant research, including studies involving school nurses and other social groups (Tzimpoulas et al. 2020; Baginska et al. 2012, 2016; Al Sari et al. 2019) and current clinical practice, a questionnaire was developed in two parts. Each question was designed for clarity and ease of response, focusing on assessing knowledge of providing first aid for various types of TDIs that may occur in a school setting. The first section included questions about demographics and training status, including sex, graduation institution, working experience as a Nurse and, more specifically, as a School Nurse, current working conditions, special dental trauma (DT) training, and DT management experience. The term “dental trauma training” was defined as any structured educational exposure, such as formal coursework, hands-on workshops, or organized continuing education sessions, focused on the management of dental trauma, rather than informal or incidental clinical experience.

The second section presented three cases of dental trauma in permanent teeth, including crown fracture, lateral luxation, and avulsion. Each case included high-resolution photographs, taken with an operating microscope, showing the trauma area in the oral cavity, along with details about the child’s age and how the TDI occurred.

In the crown fracture scenario, a 10-year-old student was described as having fallen during recess, resulting in the fracture of the right maxillary central incisor. Respondents were asked to identify whether the affected tooth was permanent or primary, to indicate whether the fractured portion could be restored, and to describe the appropriate procedures for preserving the tooth fragment until referral to a dentist. The questionnaire item referring to “liquid” storage was intentionally phrased broadly to capture respondents’ baseline understanding of suitable storage conditions, rather than to assess knowledge of specific preservation solutions.

In the lateral luxation case, a 9-year-old student sustained an injury during a Physical Education class, causing moderate lingual displacement of one mandibular central incisor with occlusal interference.

A multiple-choice question assessed the actions the school nurse would take in such a situation. Response options included reassuring the child, cleaning the affected area with saline, advising the child to bite on sterile gauze, informing the parents, referring the student to a dentist, and attempting to reposition the displaced tooth.

In the avulsion scenario, a 16-year−old boy lost his right maxillary central incisor during a physical altercation, and the avulsed tooth was recovered from the ground. Participants were asked whether they believed replantation was possible, whether they would attempt it at the site of the incident, the optimal timing for replantation, and the correct part of the tooth to handle. If immediate replantation was deemed unfeasible, additional questions explored their choice of storage medium for transporting the tooth, as well as their preferred cleaning method prior to referral.

To ensure content validity, the draft version was reviewed by a multidisciplinary panel of dentists experienced in managing dental trauma, including endodontists with a focus on dental trauma and paediatric dentists with recognized experience in managing traumatic dental injuries. These clinicians were selected based on their clinical experience, academic background, and involvement in dental traumatology teaching or research. The panel assessed the questionnaire for relevance, clarity, and comprehensiveness of the questions. Feedback was discussed collectively, and when disagreements arose, a senior consultant from the Department of Dental Traumatology provided the final decision to verify the content’s validity. The term “dental trauma specialist” has been used to specify these areas of expertise and qualifications.

### Sample size calculation

Following revisions, a sample size calculation (G*Power version 3.1.9.7, Universität Düsseldorf, Germany) was performed to determine the minimum number of necessary samples. With 2300 registered school nurses, a confidence level of 95%, and a power of 0.8, a minimum number of 87 valid questionnaires was needed. Although Power Calculation was used to determine the minimum sample size required, a probability-based sampling method was employed to produce a nationwide sample, aiming that the findings from the questionnaire can be generalized to the broader population.

### Data collection

A nationwide, cluster-based electronic survey was conducted. School districts served as clusters, and all registered school nurses within each district were invited to participate via their institutional email. Participation was voluntary, and no random selection within clusters was performed. This approach aimed to distribute the questionnaire through various channels, maximizing its reach and response rates nationwide while ensuring broader geographical representation.

### Data analysis

Data were analyzed using chi-square and Fisher’s exact tests in SPSS Statistics for Windows, version 28.0. (IBM Corp., NY, USA) to identify statistically significant differences. Descriptive statistics were used to summarize demographic characteristics and responses to the questionnaire.

Binary and logistic regression analyses were employed to determine the factors influencing decision-making at *p* < 0.05. Independent variables were selected a priori based on their clinical relevance and previous literature and included sex, graduation institution, years of experience as a nurse and as a school nurse, level of postgraduate education, working conditions, previous dental trauma (DT) training, and prior experience with DT cases at school. Dependent variables reflected decision-making in dental trauma scenarios, including willingness and ability to perform tooth replantation. Multicollinearity among independent variables was assessed using Variance Inflation Factors (VIFs). All VIF values ranged from 1.041 to 1.352, indicating no significant multicollinearity among predictors. Odds ratios (ORs) with 95% confidence intervals (CIs) were calculated to estimate the strength of associations. Statistical significance was set at *p* < 0.05, and exact *p* values are reported where applicable.

Registered school nurses in Greece (n = 2300) were reached by their institutional email within school district (cluster) and a random sample within each cluster was surveyed. A proportionate number of participants reflecting the demographics of the target population was chosen from each cluster to ensure that responses from nurses working in different school settings were acquired. The correspondence rate was set at 5.91% (*n* = 136).

## Results

### Demographic characteristics

**Fig. 1:**
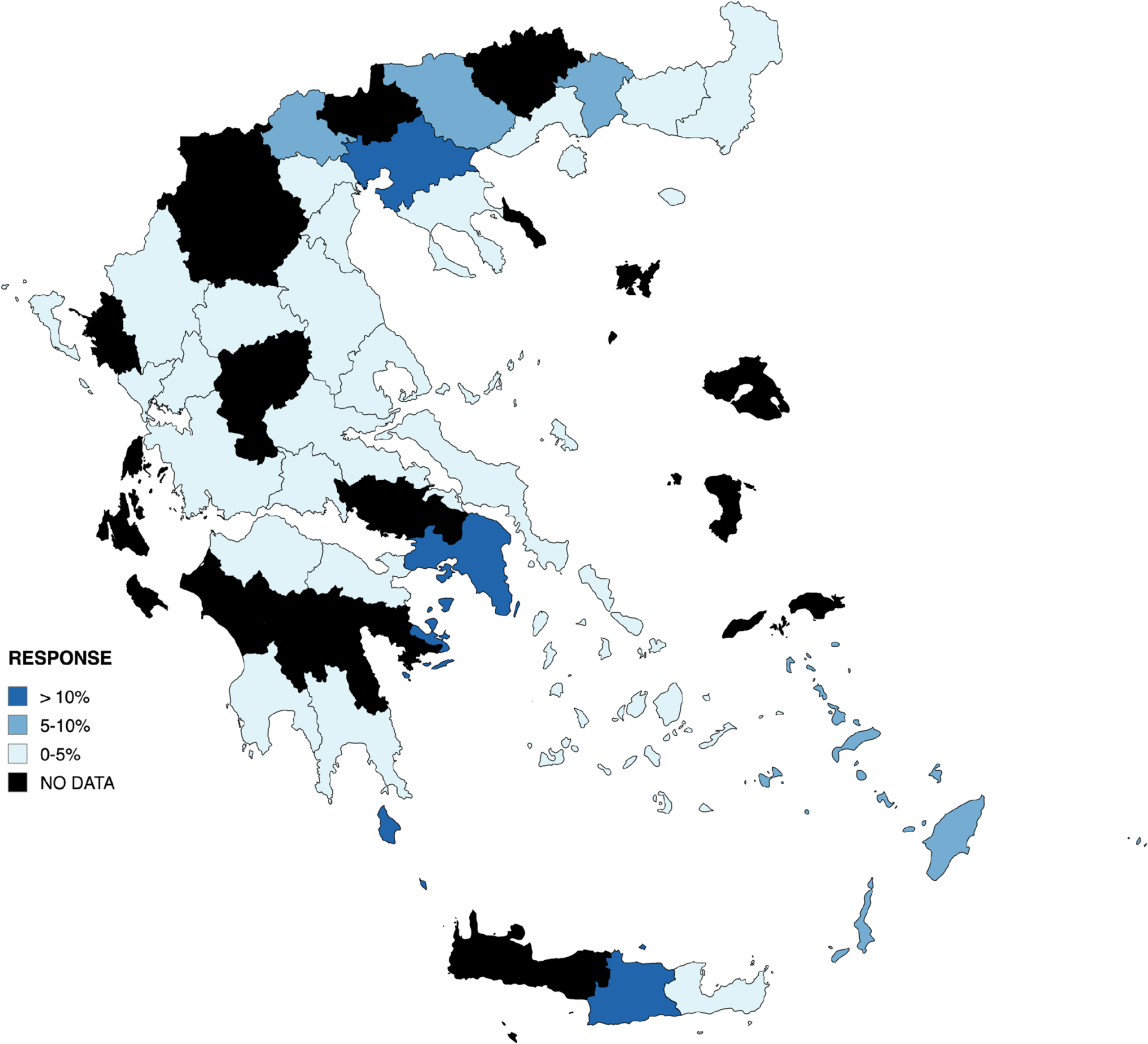
Distribution of the participants in the Greek prefectures, *n* = 136

Table 1 illustrates the demographic characteristics of the respondents, while in Fig. 1, the distribution of the participants in the Greek prefectures is depicted. 96 (70.6%) participants were enrolled in a postgraduate course or some form of postgraduate training; 47 (34.6%) respondents have completed a continuing education program on TDIs. As the main sources of information for TDI management, 35 (25.7%) included first-aid certification courses, 23 (16.9%) online resources, 16 (11.7%) informational brochures about school nursing, and only 18 (13.2%) from pre- and postgraduate studies.

**Table 1:** Demographic characteristics of the participants (*n* = 136)

Demographic characteristics		Results % (*n*)
Sex	Male	7.4% (10)
	Female	92.6% (126)
Graduation institution	University	26,5% (36)
	Technological Educational Institute	71.3% (97)
	Abroad School	2.2% (3)
Level of postgraduate education	Master	64.7% (88)
	Specialty	5.9% (8)
	PhD	1.5% (2)
	None	27.9% (38)
Working time as a nurse	0–5 years	47.8% (65)
	5–10 years	33.1% (45)
	> 10 years	19.1% (26)
Working time as a school nurse	0–5 years	77.2% (105)
	5–10 years	22.8% (31)
School	Public	98.5% (134)
	Private	1.5% (2)
School grade	Elementary	63.2% (86)
	Secondary	30.1% (41)
	High	6.6% (9)

### Knowledge regarding TDIs

78 (57.4%) respondents declared that they had experienced a dental trauma case at school; 58 (42.6%) provided emergency treatment in 1–2 cases, 17 (12.5%) in 3–4 cases, and only 3 (2.2%) in more than five dental trauma cases; 134 (98,5%) declared that they were interested in getting informed about dental trauma (Table 2). 84 (61.8%) indicated that, in case of dental trauma, they preferred to immediately call parents (*p* < 0.001). As for healthcare personnel, a paediatric dentist (61%, *n* = 83) was the preferred choice (*p* < 0.001), followed by a family dentist (22.1%, *n* = 30) and a healthcare specialist in a hospital (9.6%, *n* = 13). A dental school healthcare (2.9%, *n* = 4), general medical doctor (2.2%, *n* = 3), and endodontist (2.2%, *n* = 3) were less commonly preferred (Table 2).

**Table 2:** Knowledge and experience of the participants in TDI (*n* = 136)

TDI knowledge and experience		Results % (*n*)
Dental trauma training	No	65.4% (89)
	Yes	34.6% (47)
Have you experienced any TDI cases at school?	Yes	57.4% (78)
	No	42.6% (58)
If yes, how many TDI cases have you provided emergency treatment in your working years as a school nurse? Percentages are calculated based on participants who answered “Yes” to the previous question	1–2 Cases	42.6% (58)
	3–4 Cases	12.5% (17)
	> 5 Cases	2.2% (3)
Would you like to learn more about TDIs and their management?	Yes	98.5% (134)
	No	1.5% (2)
If a TDI occurs, whom are you calling immediately?	Parents	61.8% (84)
	Healthcare personnel	3.7% (5)
	Both	30.9% (42)
	None	0.7% (1)
	Don’t know	2.9% (4)
If a TDI occurs, who healthcare provider would you call?	Pediatric dentist	61% (83)
	Family dentist	22.1% (30)
	Hospital personnel	9.6% (13)
	Dental school personnel	2.9% (4)
	General medical doctor	2.2% (3)
	Endodontist	2.2% (3)

### Case scenario management

In the case of crown fracture, 104 (76.5%) indicated that the tooth was permanent (*p* < 0.001). Moreover, 89 (65.2%) indicated that the fragment could be used (*p* < 0.001), and 82 (60.3%) would keep the fragment in liquid (*p* < 0.001) (Table 3). In the case of lateral luxation, 129 (95.5%) would call the child’s parents as the first action. 119 (88%) would reassure the child, would rinse the area with plenty of water, and would refer to a dentist. In addition, 70 (51.5%) would recommend the child to bite a piece of gauze, while only 50 (3.7%) would attempt to reposition the tooth with their fingers.

**Table 3:** Participants’ answers regarding the crown fracture case scenario (*n* = 136)

Questions	Answers	Results % (*n*)
The fractured tooth is …	Permanent	76.5% (104)
	Primary	18.4% (25)
	Don’t know	5.1% (7)
Could the tooth fragment be used for the tooth’s restoration?	Yes	65.4% (89)
	No	16.2% (22)
	Don’t know	18.4% (25)
The fragment should be kept in …?	Liquid	60.3% (82)
	Dry	9.6% (13)
	Don’t know	30.1% (41)

Finally, for avulsion, 80 (58.8%) preferred replantation as the treatment option (*p* = 0.04). 19 respondents (14%) would perform the replantation of the tooth by themselves at the site of the accident. In terms of the appropriate timing for tooth replantation, 64 (47.1%) stated that they did not know the correct timing (p < 0.001). Meanwhile, 30 (22.1%) believed that replantation within 15 min is sufficient, while 29 (21.3%) thought that less than 1 h is clinically accepted. 99 (72.8%) participants stated that they would choose to pick up the tooth by the crown (*p* < 0.001), while only 2 (1.5%) would pick it up by the root. In addition, 29 (21.3%) were uncertain of their choice, and 6 (4.4%) considered this aspect unimportant. With respect to preservation methods, cold milk was chosen by 42 (30.9%) respondents, 29 (21.3%) chose a sterilized gauze, 25 (18.4%) chose normal saline and 24 (17.6%) participants said “don't know,” while 5 (3.7%) considered using the child’s saline solution, 4 (2.9%) preferred an antimicrobial solution, 4 also (2.9%) suggested using water. 2 (1.5%) would place it anywhere, and 1 (0.7%) thought it should be kept in the child’s mouth. Finally, 99 (72.8%) would prefer to clean the tooth (*p* < 0.001). 48 (35.3%) would use normal saline, 37 (27.2%) water, 5 (3.7%) milk, and 2 (1.5%) would select the child’s saline (Table 4).

**Table 4:** Participants’ answers regarding the avulsion case scenario (*n* = 136)

Questions	Answers	Results % (n)
Would you think of replanting the avulsed tooth?	Yes	58.8% (80)
	No	41.2% (56)
Are you able to replant it?	Yes	14% (19)
	No	86% (117)
The appropriate time for tooth replantation is …	Immediately, < 15 min	22.1% (30)
	< 60 min	21.3% (29)
	< 5 h	4.4% (6)
	< 48 h	3.7% (5)
	Time is not crucial	1.5% (2)
	Don’t know	47.1% (64)
From which part of the avulsed tooth would you hold it to pick it up?	Coronal Part	72.8% (99)
	Root	1.5% (2)
	It doesn’t matter	4.4% (6)
	Don’t know	21.3% (29)
The preferred preservation medium of the avulsed tooth would be…	Water	2.9% (4)
	Cold milk	30.9% (42)
	Normal saline	18.4% (25)
	Child’s mouth	7% (1)
	Antimicrobial solution	2.9% (4)
	A bottle full of child’s saline	3.7% (5)
	Sterilized gauge	21.3% (29)
	Anywhere	1.5% (2)
	Don’t know	17.6% (24)
Would you wash the avulsed tooth?	Yes	72.8% (99)
	No	27.2% (37)
If yes, which is the preferred washing mean? Percentages are calculated based on participants who answered “Yes” to the previous question	Water	27.2% (37)
	Antimicrobial solution	7% (1)
	Cold milk	3.7% (5)
	Normal saline	35.3% (48)
	Child’s saline	1.5% (2)
	Don’t know	4.4% (6)

### Binary and multinomial logistic regression analyses

Demographic and professional variables, including sex, graduation institution, nursing and school nursing experience, training status, working conditions, and DT training and experience, were analyzed as dependent variables in relation to independent variables reflecting DT knowledge and management, such as type of fractured tooth, fragment usability, storage conditions, avulsion replantation decisions, and preservation methods. All VIF values ranged from 1.041 to 1.352, indicating no significant multicollinearity among the predictors. The only variable that significantly influenced treatment decision was dental trauma (DT) training (*p* = 0.05). Those trained in DT were more likely to proceed with replantation than those not trained. Clinical experience or postgraduate education did not seem to have influenced this decision (*p* > 0.05) (Table 5).

**Table 5:** Binary and multinomial logistic regression analysis correlate demographic factors with responses in question regarding ability to replantation

Factor		Ability to replantation		OR (95%CI)
		Yes	No	
Gender	Male	1	9	0, 752 (0,706–081)
	Female	18	108	
Graduation	University	6	30	0, 644 (0,440–3,779)
	Technological institute	13	84	0, 341 (0,047–2,47)
	Abroad	0	3	
Postgraduate	Specialty	2	6	1,83 (0,323–10,355)
	Master	13	75	0,679 (0,206–2,235)
	PhD	0	2	2,528 (0,365–17,512)
	None	4	34	
Working time	0–5	5	60	0,54 (0,281–1,039)
	5–10	8	37	
	> 10	6	20	
School working years	0–5	15	90	1,314 (0,382–4,517)
	5–10	4	27	
School grade	Elementary	14	72	1,827 (0,678–4,925)
	Secondary	5	36	
	High	0	9	
School	Public	18	116	0,226 (0,013–3,985)
	Private	1	1	
DT training	Yes	13	34	5,289 (1,858–15,061) *
	No	6	83	
More Info	Yes	19	115	1,290 (0,433–3,838)
	No	0	2	
DT in school	Yes	13	65	1,733 (0,616–4,874)
	No	6	52	
DT cases	1 or 2	9	49	0,719 (0,257–2,008)
	3 or 4	3	14	
	> 5	1	2	

^*^p = 0.05

OR odds ratio

CI confidence interval

## Discussion

School nursing is a specialized branch of professional nursing that focuses on enhancing the well-being, academic success, and lifelong health of students. To meet this objective, school nurses possess expertise in normal development and inform others about it. They promote health and safety by fostering a healthy environment, intervene in response to actual and potential health issues, and provide case management services (AAP Council on school health 2016). Although school nurses are not expected to provide definitive treatment for TDIs, they play a pivotal role in first-aid response, including reassurance, controlling bleeding and ensuring immediate referral to a paediatric dentist, someone that the majority of the participants would first contact, as it was revealed by the present study’s results (61%). The observed responses may reflect a confident and timely approach to first-aid management; however, attributing such behaviors to personal characteristics such as maturity or composure remains interpretative and cannot be definitively inferred from the present data.

The majority of the school nurses surveyed had specialized in Emergency Nursing (70.6%), which enables them to provide effective first aid promptly. This is crucial, since the time between injury and treatment significantly affects TDI prognosis (Chaudhary et al. 2021). However, in real life, the participants would first contact parents. This highlights the nurses’ emphasis on parental communication, emotional support, and timely referral for professional care.

In the case of a crown fracture, most participants correctly identified the fractured tooth as permanent, demonstrating a higher level of awareness compared with previous studies, which reported lower accuracy among both physicians and nurses (Díaz et al. 2009; Coşkun et al. 2021). This finding suggests an overall improvement in understanding amongst the present study group. Similarly, most respondents recognized that the fractured fragment could be reused for restoration, which is consistent with the results of Al Sari et al. (2019). However, knowledge regarding the proper storage of the tooth fragment was less consistent. While most participants correctly selected a liquid medium, a notable number were uncertain or chose an inappropriate dry environment. This uncertainty reflects similar findings by Needleman et al. (2013), who observed limited understanding of fracture management among emergency department physicians.

Studies have demonstrated that milk and saliva are appropriate storage media for preserving tooth fragments following crown fractures (Shirani et al. 2011; Tewari et al. 2024), and the IADT guidelines recommend soaking the fractured fragment in saline or water for 20 min before reattachment (Bourguignon et al. 2020). Not all liquids, however, are equally suitable for fragment storage: milk and physiological saline better preserve collagen integrity and tooth structure, supporting improved bond strength upon reattachment, whereas hypotonic media such as tap water can cause swelling and disruption of collagen fibers and are associated with reduced bond strength (Sung Chul Choi 2025). Nonetheless, storage in water remains preferable to dry storage (Shirani et al. 2011; Jalannavar and Tavargeri 2018).

In the present study, school nurses appeared less likely to engage in the active management of luxation injuries and instead focused appropriately on providing first aid at the site of the accident. Most respondents reported that they would first contact the child’s parents, offer reassurance, clean the affected area with water, and refer the student to a paediatric dentist. Similar findings were reported by Díaz et al. (2009), where only a minority of participants correctly identified a luxation injury or attempted to reposition the tooth. However, repositioning of luxated teeth should be performed exclusively by dentists, who can confirm the correct positioning radiographically and stabilize the tooth using an appropriate splint (Bourguignon et al. 2020). Although immediate repositioning is recommended to improve prognosis, first-aid management by school nurses should focus on assessing the injured tooth for pain on biting, displacement, and mobility; placing a moist piece of gauze, cotton swab, or clean cotton handkerchief for the patient to bite on gently and hold in place; and arranging prompt referral to a dentist (Tewari et al. , 2024).

In case of an avulsion in permanent tooth, the primary role of school nurses lies in providing immediate first aid, preserving the avulsed tooth to maintain periodontal ligament vitality, and ensuring the child is promptly referred to a dentist. Although many respondents demonstrated awareness of appropriate handling, most correctly indicating that the tooth should be held by the crown, knowledge about the time sensitivity of replantation and the correct storage medium remained limited. Unfortunately, only 30 school nurses recognized that the tooth should ideally be replanted within 15 min, which is the recommended time frame, and many remained uncertain about the appropriate transport medium. (Andersson et al. 2020; Tewari et al, 2024). Similarly, while most would rinse the tooth, not all identified saline as the preferred solution. Notably, 14% of the participants indicated they would consider replanting the tooth themselves. This is recommended by the IADT guidelines and the IADT–ASD prevention guidelines, since immediate replantation by any adult present at the site of the injury, when feasible, is appropriate (Fouad et al. 2020; Tewari et al. 2024). Low rates in this question have been reported in comparable studies (Baginska et al. 2012, 2016; Al Sari et al. 2019). These findings mirror previous studies reporting limited preparedness before targeted education (Al Sari et al. 2019). Besides that, the guidelines clearly emphasize that the final decision regarding patient care remains primarily with the treating dentist (Fouad et al. 2020), something that is highlighted by other studies (Yeng et al. 2008). Therefore, while school nurses play a crucial role in early response and referral, their focus should remain on effective first aid, awareness of the critical time window, and rapid coordination of professional dental care.

Limited knowledge of first-aid protocols for TDIs among healthcare professionals likely reflects gaps in both undergraduate training and continuing education. Studies have shown that only a small proportion of emergency personnel and nurses have received formal instruction on TDIs or are aware of the IADT guidelines (Coşkun et al. 2021; Carrion-Ruiz et al. 2024). Similar deficiencies have been reported among medical and even dental practitioners (Tewari et al. 2021a, b; Yeng et al. 2020). Evidence indicates that targeted educational initiatives, such as workshops and focused lectures, can substantially improve school nurses’ knowledge and decision-making related to dental trauma (Al Sari et al. 2019; Baginska et al. 2012). Moreover, collaboration with dentists specializing in TDI management will be beneficial, as school nurses can consult them for first-aid guidance via video or phone calls. At the same time, a trauma record can be created before the referral (Tewari et al. 2024). In addition to emergency preparedness, preventive education should also be emphasized—school nurses play a key role in promoting the use of mouthguards and raising awareness among students, parents, and school staff about preventing TDIs during sports activities (Suganya et al. 2017; O'Connell et al. 2024). Last but not least, school nurses can utilize the Tooth SOS application (https://iadt-dentaltrauma.org/patients-toothsos/) as an easily accessible, evidence-based resource to support timely decision-making and appropriate first-aid management of TDIs in both emergency and non-emergency situations within the school setting (Tewari et al. 2024).

Integrating ongoing, interdisciplinary training and prevention strategies is essential to enhance preparedness and improve outcomes following dental trauma (Tewari et al., 2024). Literature in the field of interprofessional collaboration in oral health has revealed that early integration, along with interprofessional learning and collaboration, promotes open communication, clearer roles, and greater willingness to provide oral care. These strategies improve clinical behaviors and self-confidence. Additional research should explore expanding interprofessional opportunities to further enhance collaboration across disciplines, such as TDI management (Dsouza et al. 2019).

### Strenghts and limitations

The present study is the first to assess both the knowledge and emergency response practices related to TDIs amongst Greek school nurses, addressing a significant gap in the current literature. Uniquely, this comprehensive investigation not only examines school nurses’ general knowledge but also evaluates their management of different dental trauma categories using detailed clinical scenarios. The cross-sectional design allowed efficient data collection within a short period, while the use of an electronic questionnaire facilitated rapid distribution and response across a wide geographic area (Levin 2006; Nayak and Narayan 2019). Utilizing the EU’s EUSurvey platform ensured participant anonymity and data protection, addressing key ethical considerations. The questionnaire, based on IADT guidelines and reviewed by dentists experienced in dental trauma, incorporated realistic clinical scenarios and images, enhancing both relevance and validity. A nationwide sample with broad geographical coverage was achieved. Statistical analysis confirmed internal validity, with no evidence of multicollinearity among variables. Overall, this study not only identifies an important educational gap but also underscores the link between targeted training and effective TDI management, providing valuable guidance for future curriculum development and continuing education initiatives.

A potential limitation of this study is that, although the questionnaire underwent expert review, formal reliability testing and comprehensive validation procedures were not conducted. As is common with electronic surveys, the relatively low response rate may have affected the representativeness of the findings (Kalantzis et al. 2024). Furthermore, the voluntary nature of participation and the absence of random sampling within clusters limit the generalisability of the results (response rate: 5.91%). Nevertheless, this methodology facilitated broad national coverage and the inclusion of participants from diverse geographical regions. Accordingly, the findings should be interpreted as indicative rather than conclusive, providing useful insights for hypothesis generation and informing future research. In addition, reliance on digital distribution may have restricted participation among individuals with limited internet access or lower digital literacy. Finally, the use of the generic term “liquid” in the questionnaire may have led participants to consider suboptimal storage media, potentially resulting in an overestimation of their knowledge regarding optimal storage conditions for tooth fragments.

## Conclusions

Greek school nurses have limited capability to manage TDIs. While they are responsible for providing first aid and ensuring prompt referral in these cases, their current level of preparedness is insufficient for optimal management and often deviates from recommended guidelines.

## Supplementary Information

Below is the link to the electronic supplementary material.

Supplementary Material 1.

## Data Availability

The authors confirm that the data supporting the findings of this study are available within the article and its supplementary materials.
