# Supplementary material for: Knowledge and practices of dental trauma by Greek school nurses: a questionnaire-based cross-sectional study
Source: Eur Arch Paediatr Dent. 2026 Mar 5;27(4):1159–69. doi: 10.1007/s40368-026-01191-1 (PMC13461832; doi:10.1007/s40368-026-01191-1)
Supplement: Supplementary file 1 [file 40368_2026_1191_MOESM1_ESM.pdf]

GREEK VERSION

ΔΗΜΟΓΡΑΦΙΚΑ ΧΑΡΑΚΤΗΡΙΣΤΙΚΑ ΚΑΙ ΠΡΟΣΩΠΙΚΑ ΣΤΟΙΧΕΙΑ

1. Φύλο
- Άρρεν
- Θήλυ

2. Απόφοιτος Νοσηλευτικής Σχολής:
- Α.Ε.Ι
- πρώην Τ.Ε.Ι.
- Σχολή εξωτερικού

3. Είστε κάτοχος: (Μπορείτε να επιλέξετε παραπάνω από μια απάντηση)
- Ειδίκευσης
- Μεταπτυχιακού Διπλώματος
- Κάτοχος Διδακτορικού Διπλώματος
- κανένα από τα παραπάνω

4. Νομό στον οποίο εργάζεστε:
5. Εάν έχετε κάποια ειδίκευση, πάνω σε ποιο αντικείμενο;

6. Χρόνια Εργασίας ως Νοσηλευτής:
- 0-5 έτη
- 5-10 έτη
- >10 έτη

7. Χρόνια Εργασίας ως Σχολικός Νοσηλευτής:
- 0-5 έτη
- 5-10 έτη
- >10 έτη

8. Το σχολείο, στο οποίο εργάζεστε, είναι:
- Δημοτικό
- Γυμνάσιο
- Λύκειο

9. Το σχολείο, στο οποίο εργάζεστε, είναι:
- Δημόσιο
- Ιδιωτικό

10. Έχετε ενημερωθεί ή εκπαιδευτεί πάνω στο οδοντικό τραύμα;
- ΝΑΙ
- ΌΧΙ

11. Αν ναι, από που; (Μπορείτε να επιλέξετε παραπάνω από μια απάντηση)
- Προπτυχιακές σπουδές
- Ειδίκευση
- Μεταπτυχιακές Σπουδές
- Σεμινάρια/Ημερίδες, ιδιαίτερα Πρώτων Βοηθειών
- Ενημερωτικά φυλλάδια για τους Σχολικούς Νοσηλευτές
- Διαδίκτυο

12. Θα θέλατε να ενημερωθείτε περαιτέρω πάνω στο οδοντικό τραύμα;
- ΝΑΙ
- ΌΧΙ

13. Σας έχει τύχει περιστατικό οδοντικού τραύματος στο σχολείο;

- ΝΑΙ
- ΌΧΙ

14. Αν ναι, πόσες περιπτώσεις έχετε διαχειριστεί;
- 1-2 περιπτώσεις
- 3-4 περιπτώσεις
- >5 περιπτώσεις

ΕΡΩΤΗΣΕΙΣ ΓΕΝΙΚΟΥ ΠΕΡΙΕΧΟΜΕΝΟΥ

1. Σε περίπτωση οδοντικού τραύματος, ποιον από τους παρακάτω καλείται ΆΜΕΣΑ;
- Γονείς
- Κάποιον υγειονομικό
- Και τους δυο
- Κανέναν
- Δεν γνωρίζω

2. Σε περίπτωση οδοντικού τραύματος, με ποιον υγειονομικό θα επικοινωνούσατε;
- Γενικό ιατρό
- Υγειονομικό Προσωπικό Νοσοκομείου
- Οικογενειακό γενικό οδοντίατρο
- Υγειονομικό προσωπικό Οδοντιατρικής σχολής
- Παιδοδοντίατρο
- Ενδοδοντολόγο

ΓΝΩΣΕΙΣ ΣΧΕΤΙΚΑ ΜΕ ΤΟ ΟΔΟΝΤΙΚΟ ΤΡΑΥΜΑ

Ι.ΚΑΤΑΓΜΑ ΜΥΛΗΣ (ΈΣΠΑΣΕ ΤΜΗΜΑ ΤΟΥ ΔΟΝΤΙΟΥ ΠΟΥ ΦΑΙΝΕΤΑΙ ΜΕΣΑ ΣΤΟ ΣΤΟΜΑ):

Μαθητής 10 ετών πέφτει την ώρα του διαλείμματος και σπάζει το πρόσθιο μόνιμο άνω δόντι του:

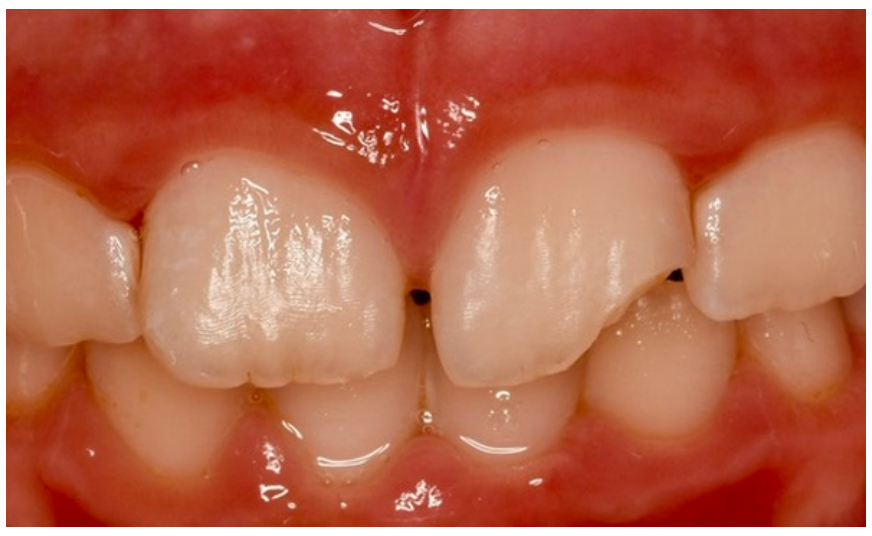

1. Το σπασμένο δόντι μπορεί να είναι:
- Νεογιλό (Παιδικό δόντι)
- Μόνιμο (Δόντι Ενηλίκων)
- Δε γνωρίζω

2. Βρίσκετε το σπασμένο κομμάτι στο έδαφος. Το κομμάτι:
- Μπορεί να χρησιμοποιηθεί
- Δεν μπορεί να χρησιμοποιηθεί πια
- Δε μπορώ να γνωρίζω

3. Εάν το σπασμένο κομμάτι μπορεί να ξαναχρησιμοποιηθεί, που φυλάσσεται;:
- Ξηρό Περιβάλλον

- Υγρό περιβάλλον
- Δε γνωρίζω

II. ΜΕΤΑΤΟΠΙΣΗ (ΤΟ ΔΟΝΤΙ ΜΕΤΑΚΙΝΗΘΗΚΕ):

Μαθήτρια 9 ετών χτυπάει στο πρόσωπο, κατά τη διάρκεια του μαθήματος Φυσικής Αγωγής. Το ένα κάτω μόνιμο πρόσθιο δόντι μετατοπίζεται μετρίως γλωσσικά με παρεμβολή στη σύγκλιση (προς τα πίσω):

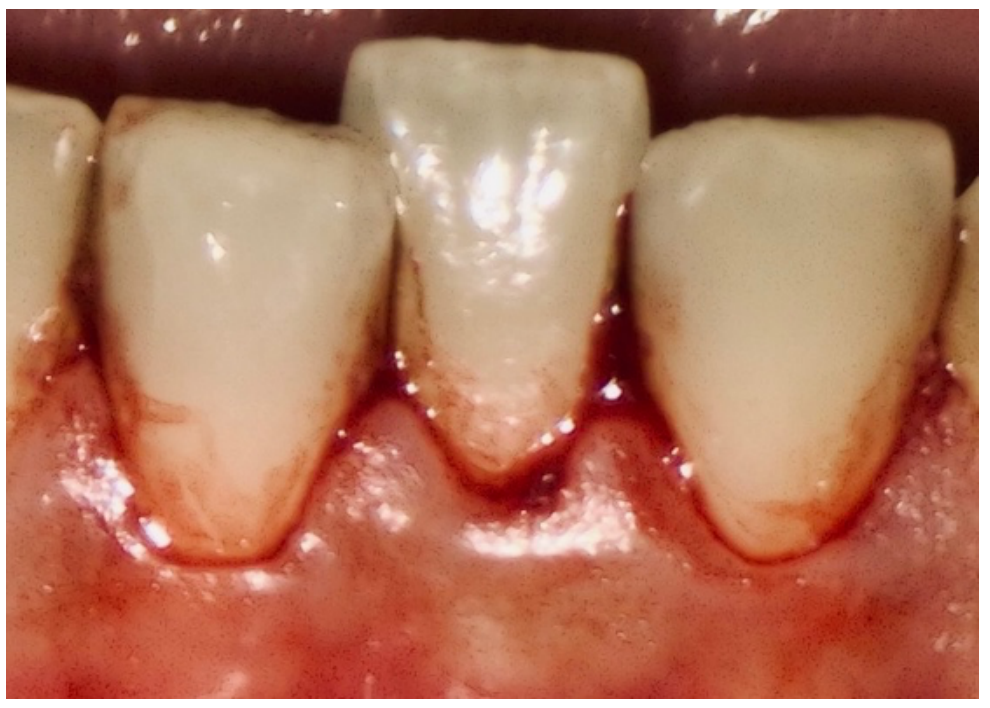

Τι θα κάνατε: (Μπορείτε να επιλέξετε παραπάνω από μια απάντηση)

- Καθησυχάζω το παιδί
- Ξεπλένω την περιοχή με άφθονο νερό
- Συμβουλεύω το παιδί να δαγκώσει μια γάζα
- Επικοινωνώ με τους γονείς του
- Παραπέμπω στον οδοντίατρο
- Προσπαθώ να επαναφέρω το δόντι στη θέση του με τα δάχτυλα μου
- Δε γνωρίζω

III. ΟΛΙΚΗ ΕΚΓΟΜΦΩΣΗ (ΤΟ ΔΟΝΤΙ ΈΧΕΙ ΒΓΕΙ ΌΛΟ ΑΠΟ ΤΟ ΣΤΟΜΑ):

Κατά τη διάρκεια αθλοπαιδιών, αγόρι 16 ετών έρχεται σε διαπληκτισμό με άλλο άτομο, με αποτέλεσμα ξυλοδαρμό και απώλεια του ενός πρόσθιου μονίμου δοντιού της άνω γνάθου. Το δόντι βρίσκεται στο έδαφος.

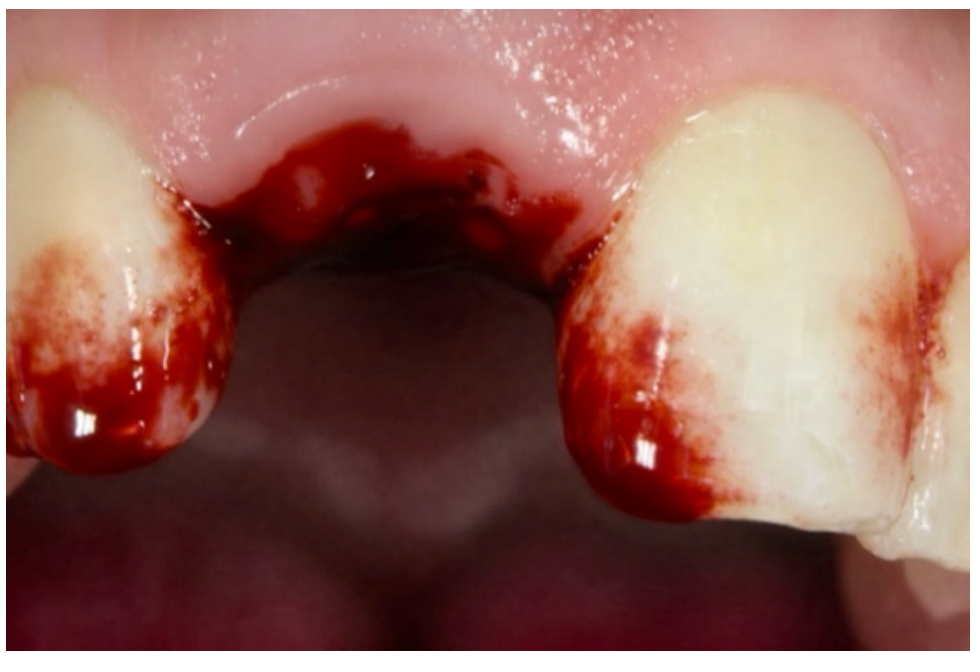

1. Μπορεί το δόντι να επανατοποθετηθεί;
- ΝΑΙ
- ΌΧΙ

2. Θα επιχειρούσατε επανατοποθέτηση του δοντιού στο χώρο του ατυχήματος;
- Ναι, θα επιχειρούσα
- Όχι, θα το παρέπεμπα

3. Εάν ναι, μέσα σε πόσο χρονικό διάστημα πρέπει να συμβεί;
- Άμεσα, εντός 15 λεπτών από τον τραυματισμό
- Εντός 1 ώρας από τον τραυματισμό
- Εντός 5 ωρών από τον τραυματισμό
- Εντός των πρώτων 48 ωρών από τον τραυματισμό
- Δεν υπάρχει περιορισμός στην ώρα
- Δεν γνωρίζω

4. Από ποιο σημείο του εκγομφωμένου δοντιού θα το πιάσετε για να το σηκώσετε;
- Από τη μύλη
- Από τη ρίζα
- Από οποιοδήποτε σημείο του δοντιού
- Δε γνωρίζω

5. Σε περίπτωση που δεν επανατοποθετηθεί και παραπέμψετε, πού θα φυλαχτεί το δόντι μέχρι το παιδί να επισκεφθεί τον αρμόδιο υγειονομικό;
- Σε νερό
- Σε κρύο γάλα
- Σε φυσιολογικό ορό
- Στο στόμα του παιδιού
- Σε αντιμικροβιακό διάλυμα
- Σε ποτήρι με το σάλιο του παιδιού
- Σε αποστειρωμένη γάζα
- Σε χαρτομάντηλο
- Μπορεί να φυλαχθεί οπουδήποτε
- Δεν γνωρίζω

6. Θα πλύνετε το δόντι πριν το παραπέμψετε ;
- ΝΑΙ
- ΌΧΙ

7. Εάν ναι, πώς θα το ξεπλένατε;
- Πλύσιμο με άφθονο κρύο νερό
- Ελαφρύ βούρτσισμα της εξωτερικής επιφάνειας με μαλακή οδοντόβουρτσα
- Πλύσιμο με αντιμικροβιακό διάλυμα
- Πλύσιμο με γάλα
- Πλύσιμο με φυσιολογικό ορό
- Πλύσιμο με το σάλιο του παιδιού
- Δε γνωρίζω

ENGLISH VERSION

DEMOGRAPHIC CHARACTERISTICS AND PERSONAL INFORMATION

1. Gender
   - Male
   - Female

2. Graduate School of Nursing:
   - University
   - Former Technological Educational Institute
   - School abroad

3. You hold: *(You may select more than one answer)*
   - Specialty / Professional specialization
   - Master's Degree
   - Doctoral Degree (PhD)
   - None of the above

4. Region/Prefecture in which you work:
   ……………………………………

5. If you have a specialty, in which field?
   ……………………………………

6. Years of work experience as a Nurse:
   - 0–5 years
   - 5–10 years
   - >10 years

7. Years of work experience as a School Nurse:
   - 0–5 years
   - 5–10 years
   - >10 years

8. The school in which you work is:
   - Primary School
   - Junior High School
   - Senior High School

9. The school in which you work is:
   - Public
   - Private

10. Have you received information or training on dental trauma?
    - YES
    - NO

11. If yes, from where? *(You may select more than one answer)*
    - Undergraduate studies
    - Specialty training
    - Postgraduate studies

- Seminars / Workshops (especially First Aid training)
- Informational leaflets for School Nurses
- Internet

12. Would you like to receive further information/training on dental trauma?
    - YES
    - NO

13. Have you encountered any incidents of dental trauma at school?
    - YES
    - NO

14. If yes, how many cases have you managed?
    - 1–2 cases
    - 3–4 cases
    - >5 cases

GENERAL KNOWLEDGE QUESTIONS

1. If a TDI occurs, whom are you calling immediately?
    - Parents
    - Healthcare Personnel
    - Both
    - None
    - Don't know

2. If a TDI occurs, who healthcare provider would you call?
    - Pediatric Dentist
    - Family Dentist
    - Hospital Personnel
    - Dental School Personnel
    - General Medical Doctor
    - Endodontist

KNOWLEDGE REGARDING DENTAL TRAUMA

I. CROWN FRACTURE *(A part of the tooth that is broken)*

A 10-year-old student falls during recess and breaks his maxillary permanent anterior tooth.

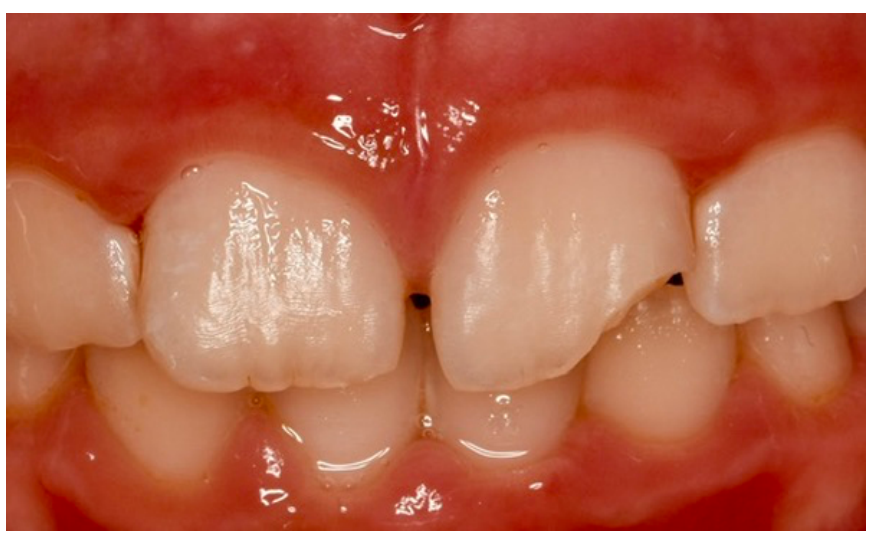

1. The fractured tooth is …
- Permanent
- Primary
- Don't know

2. You found the fractured piece of the tooth on the ground. Could the tooth fragment be used for the tooth's restoration?
- YES
- NO
- Don't know

3. If the fragment could be used for restoration, it should be kept in …?
- Liquid Environment
- Dry Environment
- Don't know

II. LATERAL LUXATION *(The tooth has been displaced from its original position)*

A 9-year-old student is hit in the face during a Physical Education class. The mandibular permanent central incisor is moderately displaced lingually (towards the back) with occlusal interference.

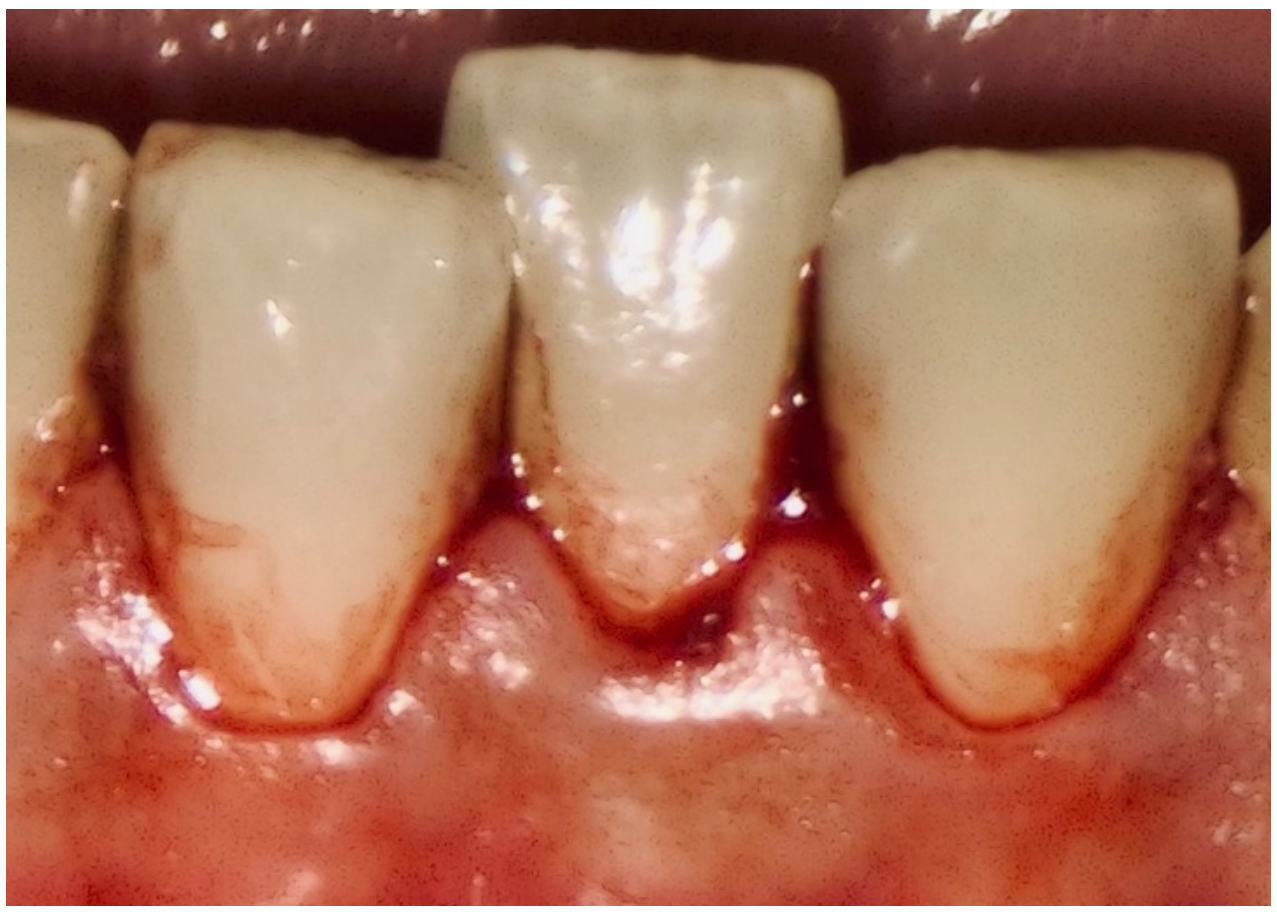

What would you do? (You may choose more than one answer)

- I reassure the child
- I rinse the area with plenty of water
- I advise the child to bite on a piece of gauze
- I contact the parents
- I refer to a dentist

• I try to reposition the tooth with my fingers
• I don't know

III. AVULSION (THE TOOTH HAS COME COMPLETELY OUT OF THE MOUTH):

During sports activities, a 16-year-old boy gets into an altercation with another person, resulting in a fight and the loss of one maxillary permanent anterior tooth. The tooth is found on the ground.

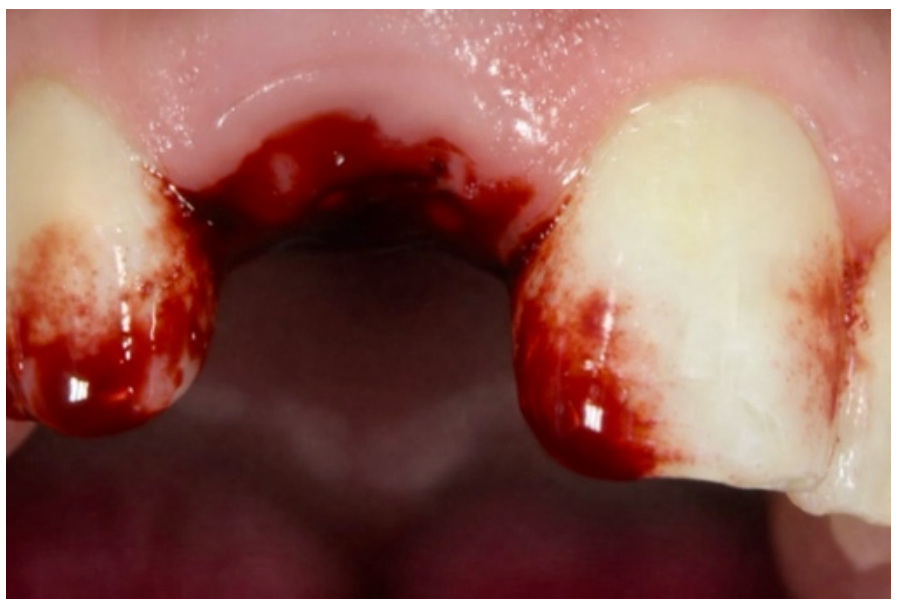

1. Would you think of replanting the avulsed tooth?

- Yes
- No

2. Are you able to replant it?

- Yes
- No

3. The appropriate time for tooth replantation is …

- Immediately, < 15min
- < 60min
- < 5h
- < 48h
- Time is not crucial
- Don't know

4. From which part of the avulsed tooth would you hold it to pick it up?

- Coronal Part
- Root
- It doesn't matter
- Don't know

5. If it is not replanted and you refer the child, the preferred preservation medium of the avulsed tooth would be…

- Water
- Cold Milk
- Normal Saline
- Child's Mouth
- Antimicrobial Solution
- A bottle full of child's saline
- Sterilized Gauge
- Anywhere
- Don't know

6. Would you wash the avulsed tooth, before the referral?

- Yes
- No

7. If yes, which is the preferred washing mean?

- Water
- Antimicrobial Solution
- Cold Milk
- Normal Saline
- Child's Saline
- Don't know
